# Signaling of Mitogenic and Metabolic Activities by Fibroblast Growth Factors

**DOI:** 10.33696/signaling.6.127

**Published:** 2025

**Authors:** Patience Salvalina Okoto, Zeina Alraawi, Thallapuranam Krishnaswamy Suresh Kumar

**Affiliations:** 1 Department of Chemistry and Biochemistry, University of Arkansas, Fayetteville, AR 72701, USA

**Keywords:** Fibroblast growth factors, Cell signaling, Mitogenic FGFs, Metabolic FGFs

## Abstract

Fibroblast growth factors are signaling molecules that play crucial roles in fundamental processes such as cell migration, proliferation, differentiation, angiogenesis, and cell survival. FGFs signal by forming a complex with tyrosine kinase FGF receptors and cofactors. It is well known that FGFs play diverse roles in different tissues and at different developmental stages due to factors such as receptors, specific FGFs, and environmental conditions such as temperature and pH. This review focuses on the actions of mitogenic and metabolic FGFs and their interactions with their receptors to trigger their designated functions.

## Introduction

Cell signaling is a process by which cells interact with each other and their environment through hormones, neurotransmitters, and growth factors that bind to specific receptors [1]. Growth factors are grouped according to their structure, function, and the type of cell or tissue that they regulate. Among the many growth factors are the fibroblast growth factor (FGF) family, transforming growth factor β (TGF-β) family, vascular endothelial growth factor (VEGF) family, epidermal growth factor (EGF) family, hepatocyte growth factor (HGF) family, platelet-derived growth factor (PDGF) family, insulin growth factor (IGF) family and neurotrophins family [2].

The fibroblast growth factor (FGF) family consists of cell signaling molecules that play major roles in cell proliferation, migration, morphogenesis, differentiation, tissue repair, and regeneration. FGF, together with other growth factors such as epidermal growth factor (EGF), and tumor necrosis factor (TNF)-α, are part of the MAP kinase pathways. These growth factors are activated by the Transforming growth factors (TGF-β). The cross-talk between these intracellular pathways is known to affect gene expression. For example, in wild-type mice with injured gut, there is dysregulation of the microbiota, which in turn leads to the upregulation of TGFβ1 expression. The upregulated TGFβ1 promotes FGF2 expression and FGF2 cross-talk with interleukin 17 (IL-17). The FGF2-IL-17 cross-talk leads to the expression of an array of repair-associated genes in intestinal epithelial cells [3].

**Figure 1:**
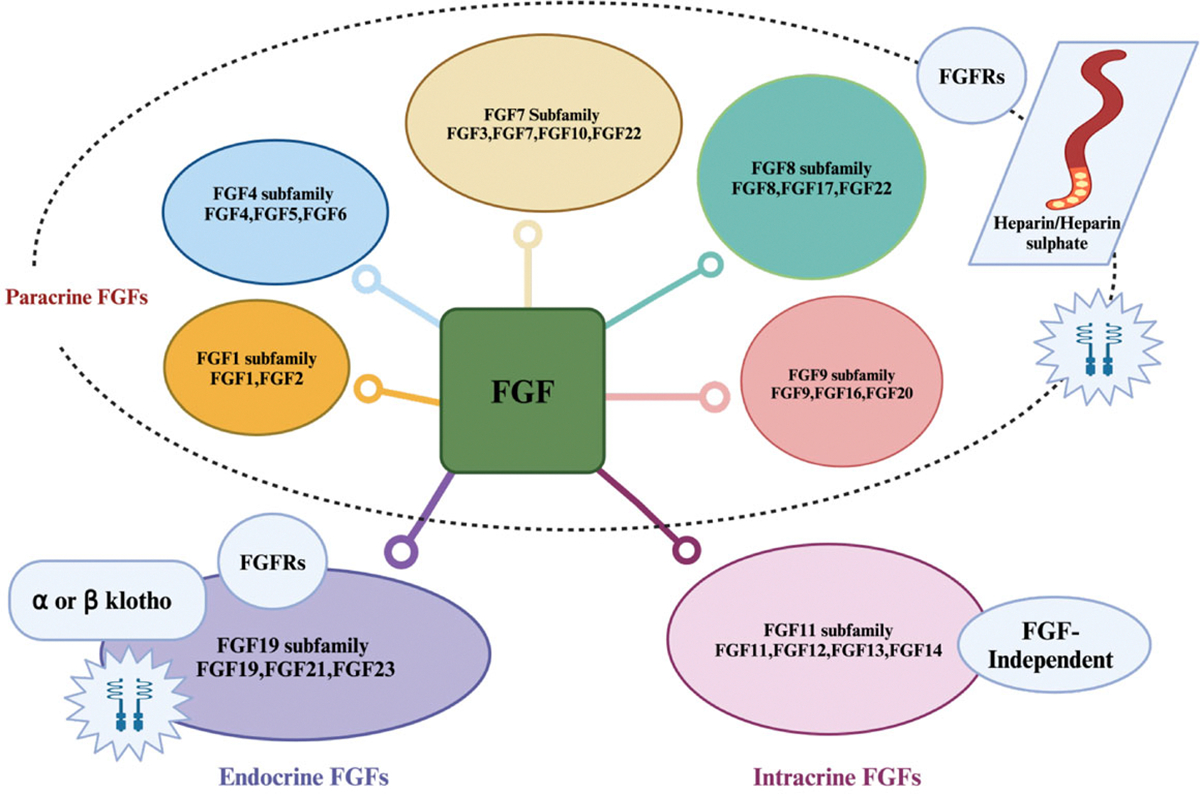
Classification of fibroblast growth factors. The figure was created with BioRender software (https://www.biorender.com)

FGFs are comprised of 22 members and are further grouped into 7 subfamilies based on their signaling events and functions. It is established that the 22 members share 13–71% sequence similarity in mammals and each subfamily possesses a wide range of biological functions [4,5]. The FGF1 subfamily comprises of FGF1 and FGF2, FGF4 subfamily comprises of FGF4, FGF5 and FGF6, FGF7 subfamily consists of FGF3, FGF7, and FGF10, FGF8 subfamily comprises FGF8, FGF17, and FGF18, FGF9 subfamily comprises of FGF9, FGF16, and FGF20, FGF11 subfamily comprises of FGF11, FGF12, FGF13, and FGF14, FGF19 comprises of FGF15/FGF19, FGF21, and FGF23 (Figure 1). FGF15 and FGF19 are orthologs with FGF15 associated with mice and FGF19 found in humans and primates [6]. Based on their mode of triggering their functions, FGFs have been divided into paracrine, intracrine, and endocrine. FGF1, FGF4, FGF7, and FGF9 subfamilies are paracrine FGFs, the FGF11 subfamily is intracrine FGFs and FGF15/19 are endocrine FGFs. Canonical FGFs also known as paracrine FGFs are tightly bound to heparin and heparin sulfate (HS) glycosaminoglycan cofactor and this association promotes FGF signaling by stabilizing the interaction of FGFs with their receptor as well as limiting diffusion through the extracellular matrix. Endocrine FGFs on the other hand have a lower binding affinity to heparin and heparin sulfate glycosaminoglycan but interact with Klotho proteins as cofactors. Intracrine FGFs are not secreted but are cofactors for voltage-gated sodium channels [7–9]. FGF signaling is triggered through the tyrosine kinase receptor, which consists of three immunoglobulin-like domains: I, II, and III. Ligand binding is specified by immunoglobin-like II and III, and the linker is located between them while immunoglobin-like I and an acidic amino acid residue (Acidic box) located between domains I and II are known to inhibit ligand binding [7,10–14].

### Mitogenic Fibroblast Growth Factors

Paracrine fibroblast growth factors include FGF1 subfamily, FGF4 subfamily, FGF7 subfamily, FGF8 subfamily, and FGF9 subfamily. These fibroblast growth factors exhibit mitogenic, angiogenic, differentiation, chemotactic, and anti-apoptotic properties. Therefore, they can function on stem cells and various cell types of mesenchymal and epithelial origins. They are also candidates for wound healing and tissue repair [15–17].

### Mitogenic Fibroblast Growth Functions via Interaction with Tyrosine Kinase FGF Receptors and Heparin

**Figure 2:**
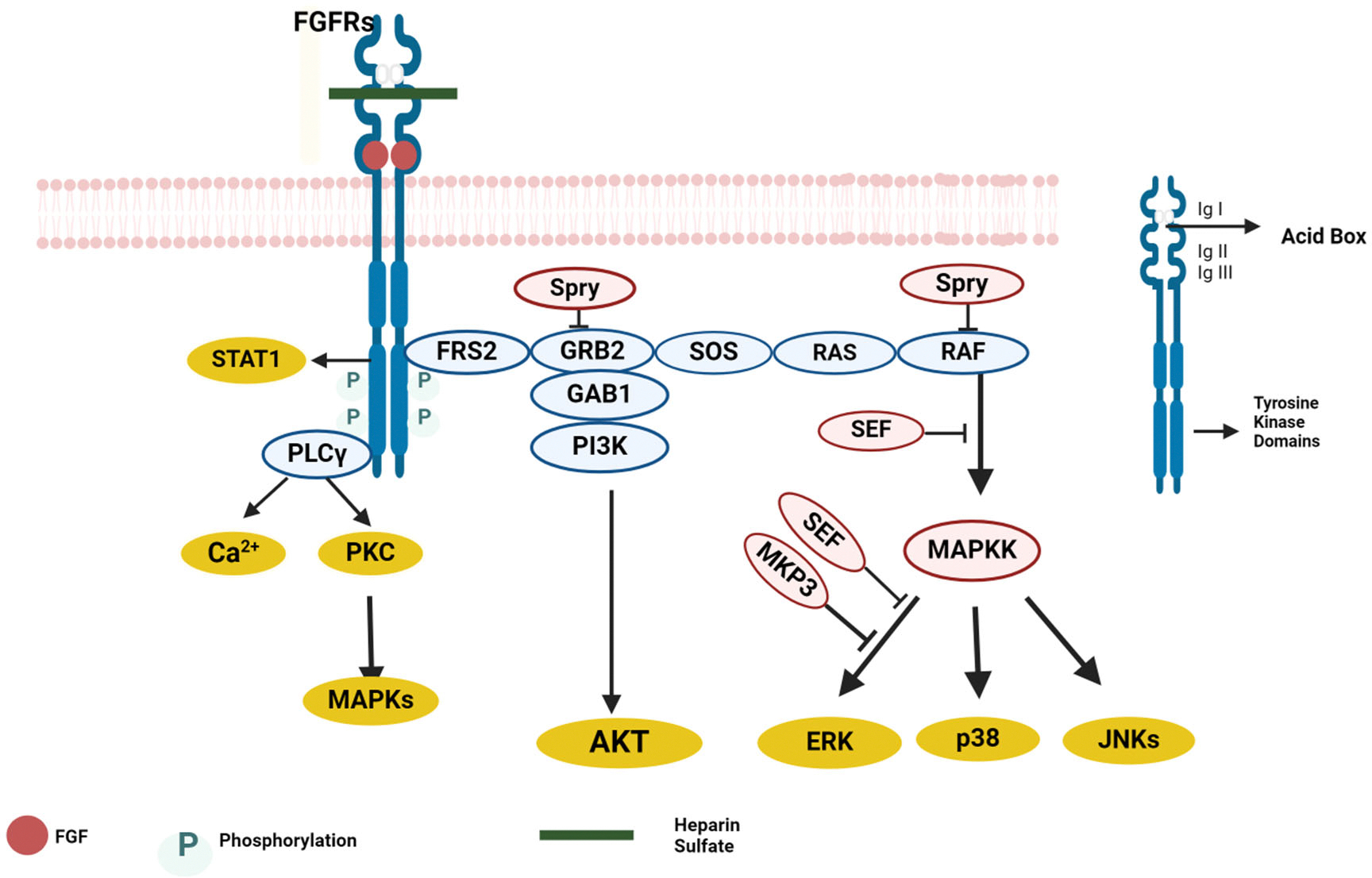
FGF signaling pathway: Classical FGFs trigger their signaling by interacting with the FGFRs using HS as a cofactor. Upon binding, the FGFR undergoes conformational changes leading to receptor dimerization and subsequent activation. Upon FGFR activation, several pathways are activated. These pathways include the FRS2-Ras-MAPK pathway, PLCγ pathway, and PIK3-AKT pathway. FGFR activation also triggers negative feedback by activating the SPRY proteins. The figure was created with BioRender software (https://www.biorender.com)

Paracrine fibroblast growth factors function locally via interaction and activation of cell surface tyrosine kinase FGF receptors (FGFRs) through a high-affinity interaction with heparin or heparan sulfate [15]. FGFRs are transmembrane proteins consisting of four genes (FGFR1, FGFR2, FGFR3 and FGFR4). Heparin-dependent FGF-FGFR binding causes receptor dimerization and trans-autophosphorylation of the tyrosine receptor. FGFRs contain an extracellular domain, a transmembrane domain (TMD), and an intracellular tyrosine kinase domain. The extracellular domain comprises three immunoglobulin (Ig)- like domains (I, II, and III), an acidic region, and a heparin-binding motif. The transmembrane domain is known to anchor the FGFs in the cell membrane and promote its dimerization. Activated Fibroblast Growth Factor Receptors (FGFR) induce the main downstream intracellular signaling pathways [Rat Sarcoma Virus (RAS)/mitogen-activated protein kinase (MAPK)], phosphatidylinositol-4,5-bisphosphate 3-kinase (PI3K)–protein kinase B (AKT), and phospholipase C gamma (PLCγ) ) and Signal Transducer and Activator of Transcription (STAT) [16] as depicted in Figure 2. Various gene expressions are controlled by RAS-MAPK signaling pathway via phosphorylation of E26 transformation-specific (ETS) transcription factors that stimulate DNA interaction and gene expressions. Phosphorylation events in embryogenesis play various physiological roles in the adult state, including regulating angiogenesis, wound healing, and organ development [17]. In contrast to the paracrine FGFs, intracrine FGFs (FGF11, FGF12, FGF13, FGF14) do not possess the N-terminal signal peptide, which is why they function as intracellular proteins [18].

Fibroblast growth factor 1 (FGF1), an acidic fibroblast growth factor, is a member of the FGF1 subfamily. FGF1 has been correlated with various biological actions such as development, angiogenesis, and adipogenesis [9]. It has been indicated that FGF1 does not have classical secretory signal peptides; instead, it is exported via direct translocation through the cell FGF1 has various functions, including regulating the cell cycle, cell differentiation, survival, and apoptosis [12]. Amongst all FGFs, FGF1 is the only isoform that can activate all FGFR splice variants [13]. The second member in the FGF1 subfamily is the basic FGF or FGF2. FGF2 plays a role in fibroblasts and other mesoderm-derived cell types, such as vascular endothelial cells, smooth muscle cells, osteoblasts, and chondrocytes. Recombinant FGF2 has medical applications in skin wound-healing processes [19]. FGF4, FGF5, and FGF6 play an essential role in various cellular processes, particularly embryonic development and tissue repair [20]. These fibroblast growth factors are involved in cell proliferation, differentiation and tissue morphogenesis [21]. FGF4 is highly expressed in the ectoderm, mesoderm, and tail bud [22]. FGF4 has multiple functions in developmental processes, such as cardiac valve leaflet formation and limb development [15]. FGF4 application is emerging as it has a unique role in oncogenesis, tumor progression, and resistance to anti-tumor therapy in multiple types of cancer [23]. FGF5 has a negative effect on controlling the hair follicle growth cycle [15]. Functional studies also reveal FGF5 overexpression in prostate cancer (PCa) and benign prostatic hyperplasia (BPH). The development of new FGF5 mitogen is upregulated after muscle injury [16,25]. FGF6 and FGF9 are known adipokines that regulate uncoupling protein 1 (UCP1) through a transcriptional network that is dissociated from brown adipogenesis and acts to modulate systemic energy metabolism. Further studies on FGF6 and FGF9 could lead to the development of therapeutic strategies to tackle obesity, type 2 diabetes, and cardiovascular disease [26].

The fibroblast growth factor 7 subfamily consists of FGF3, FGF7, FGF10, and FGF22 [17]. The members of this subfamily are secreted specifically by mesenchyme, and in the overlying epithelium, they activate the FGFR1b and FGFR2b isoforms. As a result, the signaling of these members is important to the development of essentially all organs, glands, and limbs [18].

### Metabolic FGFs

**Figure 3:**
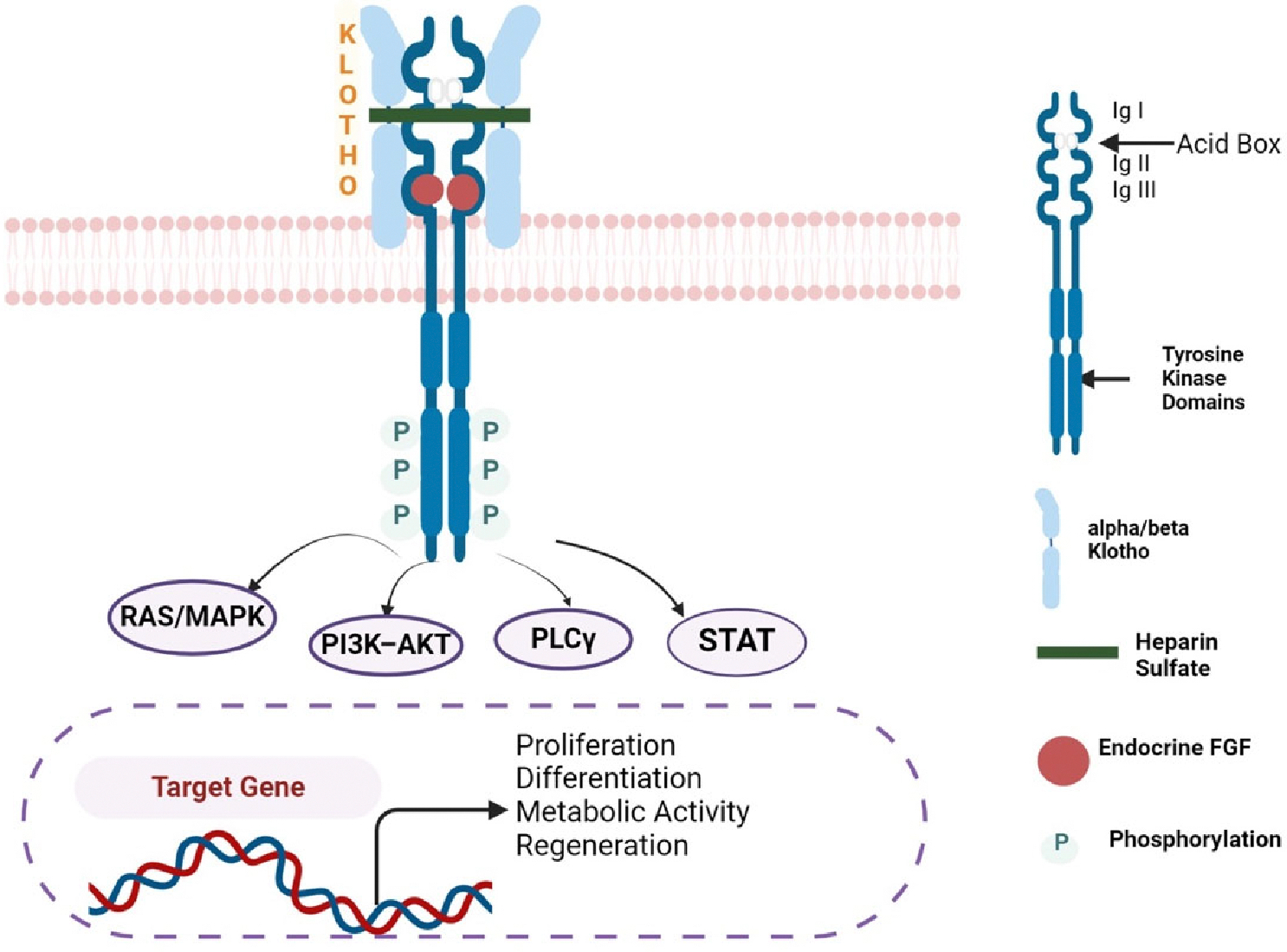
Endocrine FGF signaling: Endocrine FGF interacts with FGFR using Klotho as a cofactor. The FGF-FGFR-Klotho complex results in the activation of FGFR tyrosine kinase which then triggers the activation of RAS/MAPK, PI3K-AKT, PLCγ, and STAT pathways. The figure was created with BioRender software (https://www.biorender.com)

Metabolic FGFs regulate signaling pathways including RAS/MAPK, PI3K–AKT, PLCγ, and STAT, as shown in Figure 3. These FGFs regulate pathways that maintain the homeostasis of bile acids, lipids, glucose, energy, and minerals [27,28]. FGF15/19 triggers its metabolic effect by primarily interacting with both FGFR1 and FGFR4 and needs β-Klotho as a cofactor. FGF15/19 is released by epithelial cells in the small intestines and plays a regulatory role in bile acid, lipid, and carbohydrate metabolism [29–31]. To gain better insights into FGF15/19’s role in metabolism, studies using FGF15 knockout and liver-associated β-Klotho mice revealed abnormal glucose homeostasis and elevated gluconeogenesis levels in the fed state. Potthoff et al work on glucose metabolism, also shows that FGF15/19 inhibits hepatic gluconeogenesis [32]. Interestingly, these effects on glucose metabolism are independent of insulin activity. FGF19 mimics insulin effect by promoting protein and glycogen synthesis, independent of insulin. FGF15/19 controls bile acid synthesis by downregulating cholesterol 7α-hydroxylase gene (CYP7A1), a key rate-limiting enzyme in the process. Mice lacking FGF15, FGFR4, and β-Klotho show impaired expression of bile acid synthesis after feeding. [33–35]. Various studies demonstrate the possibility of using FGF19 for the treatment of diabetes and obesity [36].

FGF21 on the other hand exerts its activity by interacting with FGFR1c and β-Klotho. FGF21 expression is influenced by nutritional conditions (such as fasting, refeeding, and overfeeding) and the organism’s stress levels. FGF21 administration is known to reduce plasma glucose levels. However, β-Klotho or FGFR1c knockout mice did not respond to FGF21 treatment. It remains unclear whether different types of adipocytes play a role in FGF21’s glucose-lowering effects [30,37,38].

FGF23, the most recently discovered endocrine FGF, has been identified as a causative factor in tumor-induced osteomalacia, characterized by phosphate wasting. FGF23 interacts with α-Klotho and FGFR1c, FGFR3c and FGFR4. FGF23 regulates calcium homeostasis and phosphates homeostasis in the kidney and parathyroid glands. FGF23 plays a crucial role in the pathophysiology of disorders like chronic kidney disease and various bone-related conditions. Mice with an FGF23 knockout exhibit phosphate retention, which leads to imbalances in phosphate metabolism [39–43].

Abnormal signaling of FGFs are related to various diseases such as metabolic diseases, limb abnormalities, mitochondrial diseases, and cancer [36].

### Klotho Proteins

Klotho proteins are essential cofactors of endocrine FGFs, stabilizing their interaction with FGFs receptors. Klotho proteins are known to promote cognition, reduce oxidative stress, and enhance synaptic plasticity. Klotho is abundantly expressed in the kidney, as well as in the choroid plexus in the brain, and sex organs such as the ovaries, testis, and placenta [44,45].

### Expression and Activity of Klotho Proteins

The overexpression of Klotho proteins increases mice’s lifespan while mice with the Klotho knockout gene result in the acceleration of aging phenotypes [46]. The Klotho gene comprises five exons and is found in the plasma membrane and Golgi apparatus. The Klotho protein has a short intracellular domain of approximately 10 amino acids, which appears to have no known function. The extracellular domain on the other hand has two internal repeats, KL1 and KL2. Four basic amino acids serve as a linker between the two internal repeats. α-Klotho, β-klotho, and γ-Klotho are the three subtypes of the Klotho transmembrane proteins. α-Klotho and β-Klotho are vital for the formation of endocrine FGFs-FGFR complexes. α-Klotho shares 42% amino acid sequence homology with β-Klotho. The expression of β-Klotho predominantly occurs in the liver and adipose tissue while α-Klotho is predominantly expressed in the distal tubule cells. β-Klotho plays a key role in metabolic activities such as glucose uptake, bile acid synthesis, and fatty acid metabolism independent of α-Klotho. α-Klotho on the other hand regulates phosphate absorption and the activity of 1,25-dihydroxyvitamin D_3_. α and β-Klotho crystal structures have been elucidated. α-Klotho co-crystallized with FGF23 and the ligand binding domain of FGFR1c revealed that α-Klotho has a long thread termed receptor binding arm which interacts directly with FGFR1c. A groove created between α-Klotho and FGFR1c is occupied by FGF23 with a C-terminus oriented towards α-Klotho and an N-terminus oriented towards FGFR1c. Similar to α-Klotho, β-Klotho crystalized with and without FGF21 shows that β-Klotho interacts with the C-terminus of FGF21. However, certain structural regions were not able to be resolved such as the receptor binding arm [47].

### Clinical Implications of FGFs

FGF signaling plays crucial roles in metabolism, development, and homeostasis, but the malfunction of FGF/FGF receptor signaling is implicated in various human diseases such as cancer, dwarfism, chronic kidney disease (CKD), obesity, and insulin resistance [48,49]. For example, FGF19 possesses potent pharmacological benefits in administration against obesity, diabetes, and fatty liver disease. However, the involvement of FGF19 in tumorigenesis is the main challenge in translating FGF19-based pharmacotherapy from the bench to the clinic. In mice, ectopic expression of FGF19 controls hepatocyte proliferation, hepatocellular dysplasia, and neoplasia, while upregulated FGF19 expression is related to tumor progression and poor prognosis in patients with hepatocellular carcinoma (HCC) [20,50,51]. In lung diseases, there is an abnormal expression of FGFs/FGFRs. In human fetal congenital cystic adenomatoid malformation, there is a 4-fold increase in expression than the normal fetal lung. FGF1/FGFR is elevated in idiopathic pulmonary fibrosis (IPF), which is projected to lead to the pathogenesis of lung fibrosis. Due to the implications of FGFs in human diseases, various efforts are being made to dissect the structural basis underpinning metabolic and mitogenic activity.

### Future Perspectives

The FGF family has been extensively studied and revealed to be the class of proteins involved in wound healing, tissue repair, and regeneration. Since FGF proteins are prone to degradation, exploring the mechanisms behind this degradation will be crucial for realizing their full potential in therapeutic applications [52]. After understanding the FGF degradation mechanism, stable FGF variants can be designed and developed using computational and site-directed mutagenesis. Additionally, during FGF-based therapeutic applications, chemical environments, release methods, such as hydrogels and nanoparticles that enhance or maintain FGF protein stability should be investigated. The Thallapuranam group has recently developed stable FGF variants with enhanced biological activity, which have shown promise in wound healing applications [53]. An example of this stable version is a variant of FGF1, Q54P/K126N/R136E, which was attained by a 3 amino acid change on the wild-type FGF1. The wild-type FGF1 without heparin has a melting temperature (Tm) of ~41°C while the Q54P/K126N/R136E FGF1 mutant has a Tm of ~60°C in the absence of heparin. When subjected to chemical denaturation with urea, wild-type FGF1 exhibited a Cm (the concentration at which 50% of the protein unfolds) of ~1.9M, while the Q54P/K126N/R136E mutant had a Cm of ~3.97M. In another study by the same group, proline in the heparin-binding site was substituted with glutamic acid, and positively charged arginine was replaced by glutamic acid. Compared to wild-type FGF1, the P135E/R136E variant had an increased Tm of ~53°C.39 [53,54]. In another study, to investigate the role of salt bridges in FGF1 structure, charge reversal at the heparin-binding pocket was carried out by substituting aspartic acid with arginine at positions 82 and 84. After subjecting the D82R/D84R double mutant to thermal denaturation, the mutant had a Tm of ~52.6 [55]. These results reveal the enhanced stability of FGF1 using computational and site-directed mutagenesis. Since the FGF19 subfamily is involved in diverse biological activities, its members offer new avenues for treating various human metabolic diseases. For example, FGF19 plays a crucial role in regulating bile acid cycles and systemic metabolism [56,57]. Current studies indicate that the FGF21 agonist is a potential therapeutic candidate for treating type 2 diabetes and obesity [58,59]. An example is Efruxifermin (Fc-FGF21), a fusion protein of the human IgG1 Fc domain combined with human FGF21. Phase 1 studies reported individuals with type 2 diabetes showed improved glycemic control [60]. FGF23 plays a key role in regulating phosphate metabolism and is implicated in conditions such as inherited hypophosphatemia rickets, familial tumoral calcinosis, X-linked hypophosphatemia, and the progression of chronic kidney disease [59]. Exciting future prospects involving mitogenic and metabolic FGFs are awaited and with a better understanding of the mechanistic steps in their regulation will pave the way for the design of potent therapeutics against a plethora of dilapidating FGF-regulated diseases.

## References

[1] Nair A, Chauhan P, Saha B, Kubatzky KF. Conceptual Evolution of Cell Signaling. Int J Mol Sci. 2019 Jul 4;20(13):3292. 31277491 10.3390/ijms20133292 PMC6651758

[2] Dong M, Blobe GC. Role of transforming growth factor-beta in hematologic malignancies. Blood. 2006 Jun 15;107(12):4589–96. 16484590 10.1182/blood-2005-10-4169 PMC1895802

[3] Song X, Dai D, He X, Zhu S, Yao Y, Gao H, Growth Factor FGF2 Cooperates with Interleukin-17 to Repair Intestinal Epithelial Damage. Immunity. 2015 Sep 15;43(3):488–501. 26320657 10.1016/j.immuni.2015.06.024

[4] Phan P, Saikia BB, Sonnaila S, Agrawal S, Alraawi Z, Kumar TKS, The Saga of Endocrine FGFs. Cells. 2021 Sep 14;10(9):2418. 34572066 10.3390/cells10092418 PMC8465397

[5] Makarenkova HP, Hoffman MP, Beenken A, Eliseenkova AV, Meech R, Tsau C, Differential interactions of FGFs with heparan sulfate control gradient formation and branching morphogenesis. Sci Signal. 2009 Sep 15;2(88):ra55. 19755711 10.1126/scisignal.2000304 PMC2884999

[6] Lew ED, Furdui CM, Anderson KS, Schlessinger J. The precise sequence of FGF receptor autophosphorylation is kinetically driven and is disrupted by oncogenic mutations. Sci Signal. 2009 Feb 17;2(58):ra6. 19224897 10.1126/scisignal.2000021 PMC2755185

[7] Wesche J, Małecki J, Wiedłocha A, Skjerpen CS, Claus P, Olsnes S. FGF-1 and FGF-2 require the cytosolic chaperone Hsp90 for translocation into the cytosol and the cell nucleus. J Biol Chem. 2006 Apr 21;281(16):11405–12. 16495214 10.1074/jbc.M600477200

[8] Yang L, Zhou F, Zheng D, Wang D, Li X, Zhao C, FGF/FGFR signaling: From lung development to respiratory diseases. Cytokine Growth Factor Rev. 2021 Dec;62:94–104. 34593304 10.1016/j.cytogfr.2021.09.002

[9] Alam R, Mrad Y, Hammoud H, Saker Z, Fares Y, Estephan E, New insights into the role of fibroblast growth factors in Alzheimer’s disease. Mol Biol Rep. 2022 Feb;49(2):1413–27. 34731369 10.1007/s11033-021-06890-0

[10] Ornitz DM, Itoh N. The Fibroblast Growth Factor signaling pathway. Wiley Interdiscip Rev Dev Biol. 2015 May-Jun;4(3):215–66. 25772309 10.1002/wdev.176 PMC4393358

[11] Wesche J, Haglund K, Haugsten EM. Fibroblast growth factors and their receptors in cancer. Biochem J. 2011 Jul 15;437(2):199–213. 21711248 10.1042/BJ20101603

[12] König HG, Fenner BJ, Byrne JC, Schwamborn RF, Bernas T, Jefferies CA, Fibroblast growth factor homologous factor 1 interacts with NEMO to regulate NF-κB signaling in neurons. J Cell Sci. 2012 Dec 15;125(Pt 24):6058–70. 23097049 10.1242/jcs.111880

[13] Ornitz DM, Itoh N. The Fibroblast Growth Factor signaling pathway. Wiley Interdiscip Rev Dev Biol. 2015 May-Jun;4(3):215–66. 25772309 10.1002/wdev.176 PMC4393358

[14] Itoh N, Ornitz DM. Functional evolutionary history of the mouse Fgf gene family. Dev Dyn. 2008 Jan;237(1):18–27. 18058912 10.1002/dvdy.21388

[15] Sugi Y, Ito N, Szebenyi G, Myers K, Fallon JF, Mikawa T, Fibroblast growth factor (FGF)-4 can induce proliferation of cardiac cushion mesenchymal cells during early valve leaflet formation. Dev Biol. 2003 Jun 15;258(2):252–63. 12798286 10.1016/s0012-1606(03)00099-x

[16] Armand AS, Laziz I, Chanoine C. FGF6 in myogenesis. Biochim Biophys Acta. 2006 Aug;1763(8):773–8. 16875743 10.1016/j.bbamcr.2006.06.005

[17] Itoh N, Ornitz DM. Evolution of the Fgf and Fgfr gene families. Trends Genet. 2004 Nov;20(11):563–9. 15475116 10.1016/j.tig.2004.08.007

[18] Zinkle A, Mohammadi M. Structural Biology of the FGF7 Subfamily. Front Genet. 2019 Feb 12;10:102. 30809251 10.3389/fgene.2019.00102 PMC6379346

[19] Itoh N, Ornitz DM. Functional evolutionary history of the mouse Fgf gene family. Dev Dyn. 2008 Jan;237(1):18–27. 18058912 10.1002/dvdy.21388

[20] Xie Y, Su N, Yang J, Tan Q, Huang S, Jin M, FGF/FGFR signaling in health and disease. Signal Transduct Target Ther. 2020 Sep 2;5(1):181. 32879300 10.1038/s41392-020-00222-7 PMC7468161

[21] Itoh N, Ornitz DM. Fibroblast growth factors: from molecular evolution to roles in development, metabolism and disease. J Biochem. 2011 Feb;149(2):121–30. 20940169 10.1093/jb/mvq121 PMC3106964

[22] Kosaka N, Sakamoto H, Terada M, Ochiya T. Pleiotropic function of FGF-4: its role in development and stem cells. Dev Dyn. 2009 Feb;238(2):265–76. 18792115 10.1002/dvdy.21699

[23] Levine KM, Ding K, Chen L, Oesterreich S. FGFR4: A promising therapeutic target for breast cancer and other solid tumors. Pharmacol Ther. 2020 Oct;214:107590. 32492514 10.1016/j.pharmthera.2020.107590 PMC7494643

[24] Stangis MM, Colah AN, McLean DT, Halberg RB, Collier LS, Ricke WA. Potential roles of FGF5 as a candidate therapeutic target in prostate cancer. American Journal of Clinical and Experimental Urology. 2023;11(6):452–66. 38148937 PMC10749387

[25] Cai Q, Wu G, Zhu M, Ge H, Xue C, Zhang Q, FGF6 enhances muscle regeneration after nerve injury by relying on ERK1/2 mechanism. Life Sci. 2020 May 1;248:117465. 32105707 10.1016/j.lfs.2020.117465

[26] Shamsi F, Xue R, Huang TL, Lundh M, Liu Y, Leiria LO, FGF6 and FGF9 regulate UCP1 expression independent of brown adipogenesis. Nat Commun. 2020 Mar 17;11(1):1421. 32184391 10.1038/s41467-020-15055-9 PMC7078224

[27] Farooq M, Khan AW, Kim MS, Choi S. The Role of Fibroblast Growth Factor (FGF) Signaling in Tissue Repair and Regeneration. Cells. 2021 Nov 19;10(11):3242. 34831463 10.3390/cells10113242 PMC8622657

[28] Katoh M Fibroblast growth factor receptors as treatment targets in clinical oncology. Nat Rev Clin Oncol. 2019 Feb;16(2):105–22. 30367139 10.1038/s41571-018-0115-y

[29] Guan D, Zhao L, Chen D, Yu B, Yu J. Regulation of fibroblast growth factor 15/19 and 21 on metabolism: in the fed or fasted state. J Transl Med. 2016 Mar 1;14:63. 26931208 10.1186/s12967-016-0821-0 PMC4774037

[30] Kurosu H, Choi M, Ogawa Y, Dickson AS, Goetz R, Eliseenkova AV, Tissue-specific expression of betaKlotho and fibroblast growth factor (FGF) receptor isoforms determines metabolic activity of FGF19 and FGF21. J Biol Chem. 2007 Sep 14;282(37):26687–95. 17623664 10.1074/jbc.M704165200 PMC2496965

[31] Lin BC, Wang M, Blackmore C, Desnoyers LR. Liver-specific activities of FGF19 require Klotho beta. J Biol Chem. 2007 Sep 14;282(37):27277–84. 17627937 10.1074/jbc.M704244200

[32] Potthoff MJ, Boney-Montoya J, Choi M, He T, Sunny NE, Satapati S, FGF15/19 regulates hepatic glucose metabolism by inhibiting the CREB-PGC-1α pathway. Cell Metab. 2011 Jun 8;13(6):729–38. 21641554 10.1016/j.cmet.2011.03.019 PMC3131185

[33] Somm E, Jornayvaz FR. Fibroblast Growth Factor 15/19: From Basic Functions to Therapeutic Perspectives. Endocr Rev. 2018 Dec 1;39(6):960–89. 30124818 10.1210/er.2018-00134

[34] Kir S, Beddow SA, Samuel VT, Miller P, Previs SF, Suino-Powell K, FGF19 as a postprandial, insulin-independent activator of hepatic protein and glycogen synthesis. Science. 2011 Mar 25;331(6024):1621–4. 21436455 10.1126/science.1198363 PMC3076083

[35] Sinha J, Chen F, Miloh T, Burns RC, Yu Z, Shneider BL. β-Klotho and FGF-15/19 inhibit the apical sodium-dependent bile acid transporter in enterocytes and cholangiocytes. American Journal of Physiology-Gastrointestinal and Liver Physiology. 2008 Nov;295(5):G996–1003. 18772362 10.1152/ajpgi.90343.2008 PMC2584833

[36] Shen L, Li Y, Zhao H. Fibroblast growth factor signaling in macrophage polarization: impact on health and diseases. Front Immunol. 2024 Jun 19;15:1390453. 38962005 10.3389/fimmu.2024.1390453 PMC11219802

[37] Martínez-Garza Ú, Torres-Oteros D, Yarritu-Gallego A, Marrero PF, Haro D, Relat J. Fibroblast Growth Factor 21 and the Adaptive Response to Nutritional Challenges. Int J Mol Sci. 2019 Sep 21;20(19):4692. 31546675 10.3390/ijms20194692 PMC6801670

[38] Rydén M Fibroblast growth factor 21: an overview from a clinical perspective. Cellular and Molecular Life Sciences. 2009 Jul;66:2067–73. 19277467 10.1007/s00018-009-0003-9 PMC11115664

[39] Ito N, Hidaka N, Kato H. The pathophysiology of hypophosphatemia. Best Pract Res Clin Endocrinol Metab. 2024 Mar;38(2):101851. 38087658 10.1016/j.beem.2023.101851

[40] ADHR Consortium. Autosomal dominant hypophosphataemic rickets is associated with mutations in FGF23. Nat Genet. 2000 Nov;26(3):345–8. 11062477 10.1038/81664

[41] Shimada T, Mizutani S, Muto T, Yoneya T, Hino R, Takeda S, Cloning and characterization of FGF23 as a causative factor of tumor-induced osteomalacia. Proc Natl Acad Sci U S A. 2001 May 22;98(11):6500–5. 11344269 10.1073/pnas.101545198 PMC33497

[42] Ito N, Hidaka N, Kato H. Acquired Forms of Fibroblast Growth Factor 23-Related Hypophosphatemic Osteomalacia. Endocrinology and Metabolism. 2024 Apr 1;39(2):255–61. 38467164 10.3803/EnM.2023.1908 PMC11066443

[43] Hu MC, Shiizaki K, Kuro-o M, Moe OW. Fibroblast growth factor 23 and Klotho: physiology and pathophysiology of an endocrine network of mineral metabolism. Annu Rev Physiol. 2013;75:50333. 10.1146/annurev-physiol-030212-183727 PMC3770142 23398153

[44] Choy C, Kim SH. Biological actions and interactions of anosmin-1. Front Horm Res. 2010;39:78–93. 20389087 10.1159/000312695

[45] Kuro-o M Klotho in health and disease. Current Opinion in Nephrology and Hypertension. 2012 Jul 1;21(4):362–8. 22660551 10.1097/MNH.0b013e32835422ad

[46] Kim JH, Hwang KH, Park KS, Kong ID, Cha SK. Biological Role of Anti-aging Protein Klotho. J Lifestyle Med. 2015 Mar;5(1):1–6. 26528423 10.15280/jlm.2015.5.1.1 PMC4608225

[47] Chen G, Liu Y, Goetz R, Fu L, Jayaraman S, Hu MC, α-Klotho is a non-enzymatic molecular scaffold for FGF23 hormone signalling. Nature. 2018 Jan 25;553(7689):461–6. 29342138 10.1038/nature25451 PMC6007875

[48] Brown LM, Ekert PG, Fleuren EDG. Biological and clinical implications of FGFR aberrations in paediatric and young adult cancers. Oncogene. 2023 Jun;42(23):1875–88. 37130917 10.1038/s41388-023-02705-7 PMC10244177

[49] Xie Y, Su N, Yang J, Tan Q, Huang S, Jin M, FGF/FGFR signaling in health and disease. Signal Transduction and Targeted Therapy. 2020 Sep 2;5(1):181. 32879300 10.1038/s41392-020-00222-7 PMC7468161

[50] Ahn SM, Jang SJ, Shim JH, Kim D, Hong SM, Sung CO, Genomic portrait of resectable hepatocellular carcinomas: implications of RB1 and FGF19 aberrations for patient stratification. Hepatology. 2014 Dec;60(6):1972–82. 24798001 10.1002/hep.27198

[51] Nicholes K, Guillet S, Tomlinson E, Hillan K, Wright B, Frantz GD, A mouse model of hepatocellular carcinoma: ectopic expression of fibroblast growth factor 19 in skeletal muscle of transgenic mice. Am J Pathol. 2002 Jun;160(6):2295–307. 12057932 10.1016/S0002-9440(10)61177-7 PMC1850847

[52] Buchtova M, Chaloupkova R, Zakrzewska M, Vesela I, Cela P, Barathova J, Instability restricts signaling of multiple fibroblast growth factors. Cellular and Molecular Life Sciences. 2015 Jun;72:2445–59. 25854632 10.1007/s00018-015-1856-8 PMC11113989

[53] Agrawal S, Govind Kumar V, Gundampati RK, Moradi M, Kumar TKS. Characterization of the structural forces governing the reversibility of the thermal unfolding of the human acidic fibroblast growth factor. Sci Rep. 2021 Aug 2;11(1):15579. 34341408 10.1038/s41598-021-95050-2 PMC8329156

[54] Davis JE, Alghanmi A, Gundampati RK, Jayanthi S, Fields E, Armstrong M, Probing the role of proline– 135 on the structure, stability, and cell proliferation activity of human acidic fibroblast growth factor. Archives of Biochemistry and Biophysics. 2018 Sep 15;654:115–25. 30031837 10.1016/j.abb.2018.07.017 PMC6152824

[55] Davis JE, Gundampati RK, Jayanthi S, Anderson J, Pickhardt A, Koppolu BP, Effect of extension of the heparin binding pocket on the structure, stability, and cell proliferation activity of the human acidic fibroblast growth factor. Biochem Biophys Rep. 2017 Dec 22;13:45–57. 29556563 10.1016/j.bbrep.2017.12.001 PMC5857160

[56] Ryan KK, Kohli R, Gutierrez-Aguilar R, Gaitonde SG, Woods SC, Seeley RJ. Fibroblast growth factor-19 action in the brain reduces food intake and body weight and improves glucose tolerance in male rats. Endocrinology. 2013 Jan;154(1):9–15. 23183168 10.1210/en.2012-1891 PMC3529386

[57] Morton GJ, Matsen ME, Bracy DP, Meek TH, Nguyen HT, Stefanovski D, FGF19 action in the brain induces insulin-independent glucose lowering. J Clin Invest. 2013 nov;123(11):4799–808. 24084738 10.1172/JCI70710 PMC3809800

[58] Itoh N FGF21 as a Hepatokine, Adipokine, and Myokine in Metabolism and Diseases. Front Endocrinol (Lausanne). 2014 Jul 7;5:107. 25071723 10.3389/fendo.2014.00107 PMC4083219

[59] Zhang F, Yu L, Lin X, Cheng P, He L, Li X, Minireview: Roles of Fibroblast Growth Factors 19 and 21 in Metabolic Regulation and Chronic Diseases. Mol Endocrinol. 2015 Oct;29(10):1400–13. 26308386 10.1210/me.2015-1155 PMC4588730

[60] Kaufman A, Abuqayyas L, Denney WS, Tillman EJ, Rolph T. AKR-001, an Fc-FGF21 Analog, Showed Sustained Pharmacodynamic Effects on Insulin Sensitivity and Lipid Metabolism in Type 2 Diabetes Patients. Cell Rep Med. 2020 Jul 21;1(4):100057. 33205064 10.1016/j.xcrm.2020.100057 PMC7659583

